# Aging and GATA3-positive macrophages

**DOI:** 10.18632/aging.101929

**Published:** 2019-04-24

**Authors:** Behrooz G. Sharifi, Mingjie Yang, Prediman K. Shah

**Affiliations:** 1 Oppenheimer Atherosclerosis Research Center, Cedars Sinai Smidt Heart Institute, Cedars-Sinai Medical Center, Los Angeles, CA 90048, USA

**Keywords:** macrophage, inflammation, subset, tissue repair, myocardial infarction

Cardiovascular disease is currently recognized as the leading cause of morbidity and mortality in the adult population worldwide, with an estimated projection of 23.3 million yearly deaths attributable to these disorders by the year 2030 [1]. Although numerous studies in the cardiovascular field have considered humans, both young and old, there remain many unanswered questions about the contribution of genetic pathways in the regulation of aging in model organisms that also influence cardiovascular aging. Chronic inflammation is believed to contribute to the pathogenesis of many age-related diseases including cardiovascular disease.

Chronic inflammation, particularly from activation of innate immunity, is highly sensitive to changes in the tissue environment that is associated with aging. The immune cell type that is particularly influenced by changes in its microenvironment is the monocyte/macrophage. These cells display a high level of plasticity and heterogeneity in response to their environmental cues. For example, based on the response of cultured macrophages to treatment with IL-4 or interferon γ, cells have been proposed to polarize to either M2 or M1 phenotypes, respectively. Although the M1-M2 polarization concept is useful in describing the two extremes of macrophage phenotypes, the concept does not accurately recapitulate the complex response of cells to their driving tissue microenvironment in vivo.

In most animal models of tissue injury including the myocardial injury, the first phase involves infiltration of pro-inflammatory Ly6C^hi^ monocytes followed by a decline in their numbers with a concomitant increase in the anti-inflammatory/reparative Ly6C^lo^ cell subset. A timely resolution of each step in the recruitment of these cells is crucial since exuberant and prolonged inflammation and/or fibrosis may be deleterious and determines whether progression to cardiac tissue repair or heart failure occurs.

The plasticity of monocytes/macrophages are determined by the constellation of transcription factors that are activated and expressed in response to environmental cues. To understand the role of GATA3 transcription factor in the pathogenesis of cardiac diseases, we generated myeloid-specific GATA3 knockout mice and found that their cardiac function is significantly improved in response to ischemia or pressure overload compared with the GATA3 sufficient control group [2]. Analysis of the profile of monocytes/macrophages in vivo revealed that GATA3-positive macrophages are not found in the healthy adult tissue, and the specific ablation of the GATA3 gene in myeloid cells had no effect on the profile of myeloid cells in the blood circulation or myocardium under the steady state conditions. In the stetting of an MI, however, the deficiency of GATA3-positive macrophages led to a significant improvement of cardiac function compared with the GATA3 sufficient control group. This improvement was found to be associated with the presence of many Ly6C^hi^ pro-inflammatory macrophages, but, few “anti-inflammatory/reparative” Ly6C^lo^ macrophages.

This was unexpected because the prevailing hypothesis is that controlling the pro-inflammatory pathways may improve cardiac function. Our data suggest that exuberant repair, rather than unrestrained inflammation, may contribute to the excessive and maladaptive remodeling of the myocardium in the post-MI setting. The notion that the presence of excessive “reparative/anti-inflammatory” macrophage subset may be a culprit in the maladaptive remodeling of the myocardium is supported by recent studies. The anti-inflammatory IL-10 cytokine, which is produced by reparative macrophages, has been recently shown to promote cardiac fibrosis and myocardial stiffness in diastolic dysfunction compared with the control [3]. In the pressure overload setting, the number of “reparative/anti-inflammatory” Ly6C^lo^ macrophages, but not the level of pro-inflammatory Ly6C^hi^ cell subset, was found to be responsible for the maladaptive remodeling of the pressure overloaded heart [4]. In the human heart, M2 macrophages are associated with cardiac fibrosis in the ischemic heart [5]. In an experimental atherosclerosis study, it was shown that blocking the pro-inflammatory cytokine IL-1β exacerbated lesion development [6]. Finally, the frequency of Ly6C^lo^ macrophage subset, but not Ly6C^hi^ subset, was reported to be associated with the fibrosis phase of the lung [7]. Thus, controlling the reparative/anti-inflammatory pathways, rather than pro-inflammatory signaling, may mitigate development of cardiovascular and possibly other fibroproliferative diseases.

Extensive evidence suggests that the aging heart undergoes fibrotic remodeling [8]. Although targeting of pro-inflammatory pathways is thought to be an important strategy to control excessive tissue fibrosis, numerous anti-inflammatory drugs including corticosteroids have been found to have little or no therapeutic benefit in idiopathic pulmonary fibrosis or other fibrotic diseases. Our data suggest that GATA3-positive macrophages, which presumably display an M2 phenotype, are highly fibrogenic. It is therefore possible that targeting a subset of inflammatory cells, rather than global inflammation, may be a useful therapeutic strategy to control fibrotic diseases associated with aging.
